# Physical Activity and Eating Habits of Polish Navy Soldiers and Border Guard Officers—A Comparative Analysis

**DOI:** 10.3390/nu16223842

**Published:** 2024-11-09

**Authors:** Andrzej Tomczak, Anna Anyżewska, Tomasz Lepionka, Izabela Bolczyk, Tomasz Grudniewski, Iwona Gładysz, Artur Kruszewski, Jerzy Bertrandt

**Affiliations:** 1 Institute of Human Sciences, WSB Merito University in Torun, 87-100 Torun, Poland; 2 School of Medical & Health Sciences, University of Economics and Human Sciences, 01-043 Warsaw, Poland; a.anyzewska@vizja.pl; 3 Laboratory of Next Generation Sequencing, Biological Threats Identification and Countermeasure Centre, Military Institute of Hygiene and Epidemiology, 01-163 Pulawy, Poland; tomasz.lepionka@wihe.pl; 4 Medical Department of the Technology and Supply Office of the Border Guard Headquarters, 00-514 Warsaw, Poland; izabela.bolczyk@strazgraniczna.pl; 5 Department of Orthopedics and Traumatology, The 7th Naval Hospital, 80-305 Gdańsk, Poland; tomek_doc@op.pl; 6 Faculty of Health Sciences, John Paul II University, 21-500 Biała Podlaska, Poland; i.gladysz@dyd.akademiabialska.pl; 7 Department of Individual Sports, Józef Piłsudski University of Physical Education in Warsaw, Marymoncka 34 St., 00-968 Warsaw, Poland; artur.kruszewski@awf.edu.pl; 8 Faculty of Economic Sciences, John Paul II University, 21-500 Biala Podlaska, Poland; jwbertrandt@gmail.com

**Keywords:** physical activity, IPAQ, eating habits, soldiers, border guard officers, uniformed services

## Abstract

Background: Physical fitness and a healthy body mass are important predictors of a good performance of military tasks. The purpose of this study was to assess the physical activity level and nutrition, in terms of the frequency of consumption of individual food groups, of Polish Navy soldiers and Maritime Unit of the Border Guard officers. Materials and Methods: This study was conducted on 131 Polish Navy soldiers aged 33.1 ± 6.3 years old and 132 Maritime Unit of the Border Guard officers aged 38.6 ± 5.5 years old. In order to carry out research on physical activity, the International Physical Activity Questionnaire was used. Eating habits were assessed based on a Food Frequency Questionnaire for 61 groups of food products. Results: Over 80% of the soldiers and officers studied indicated high levels of physical activity. Only 8.2% of soldiers and 4.3% of officers indicated a low level of physical activity. Of the 61 food product groups analyzed, significant differences were found in the frequency of consumption of 17 products. These differences concerned almost all groups (except dairy products and eggs). Conclusions: To summarize, soldiers and officers presented high and moderate levels of physical activity. Taking into account the demonstrated frequent consumption of high-energy and high-fat products, such as sausages and red meat, as well as margarine, mayonnaise, and sugar, with the low frequency of fruit and vegetable consumption in both studied groups, it is advisable to conduct training among soldiers and officers in the field of health education.

## 1. Introduction

One of the most significant requirements for soldiers or officers of uniformed formations to perform their professional work (completing tasks) is maintaining a high level of physical fitness. It has been this way since time immemorial, and it cannot be changed despite the considerable technification of equipment, adding electronics to it and implementing modern information technology. The only shift can be noticed in the preferred motor skills of a soldier/officer, from strength and endurance toward coordination motor abilities [1]. Proper nutrition plays a key role in the overall health and performance of military personnel, especially military personnel working in difficult conditions with increased physical demands. Therefore, physical activity is closely related to an appropriate nutrition model based on food rations, the energy and nutritional value of which should cover the energy and nutritional needs of soldiers and officers of the Border Guard resulting from training and service processes. This can only be achieved by rational nutrition and delivering food rations providing an amount of energy that is appropriate to the soldiers and officers’ needs, as well as a variety of food products containing all the necessary nutrients.

Armed forces are interested in wide-ranging scientific research related to the physical fitness and physical activity (PA) of soldiers, as well as planning and implementing their nutrition.

This can be demonstrated by the fact that many international and national conferences are held on the aforementioned topic [2,3,4,5]. In the case of a soldier/officer, this is proved by their participation in periodic physical fitness tests, which may lead to removal from duty if failed [6]. Physical fitness can be achieved through participating in various forms of PA. In general terms, it can be established that the intensity of physical activity, its duration, and type translate into the overall level of physical fitness. There are both utilitarian and health-related benefits of engaging in physical activity for a soldier/officer. Currently, the World Health Organization (WHO) recommends that adults (18–64 years old) should perform physical activity for 150–300 min per week (for moderate intensity) or 75–50 min (for high intensity) [7]. In the literature, a lot of attention has been given to assessing the level of physical activity of soldiers and officers of other services [8,9]. In Poland, a particularly large number of articles concerned with physical activity and nutritional status were published in a Special Issue following the 2016–2020 research project of the National Health Program [10]. After conducting a study using the International Physical Activity Questionnaire (IPAQ), Tomczak et al. [10] demonstrated that 81.8% of all Air Force soldiers had a high level of PA, 10.2% had a moderate level, and 8.0% had a low level. The same study also found that flight crews indicated a high level of physical activity, with 80.9% engaging in PA, while a moderate level was indicated by 11.9% and low by 7.2%. In another study, Maculewicz et al. found the following levels of PA in airborne battalion soldiers: high at 85.4%, moderate at 12.2%, and low at 2.4% [11]. On the other hand, it is difficult to find publications pertaining to research from other countries with the use of the IPAQ. It seems that in other armies around the world, this research tool is not used. One of the few papers that have been found is an article written by Finnish authors who assessed the PA level of recruits using the IPAQ tool prior to undertaking the study [12].

Also, the issue of proper nutrition for soldiers regarding the allocation of food rations and their implementation is the subject of many studies conducted in various parts of the Armed Forces. In the first phase, this research involves finding out the energy and nutritional needs of soldiers resulting from their training processes, considering the nature of military service and the specificity of the unit and type of armed force. In the second phase, food rations are planned and assessed [13].

In this article, the authors aim at drawing attention to the level of PA of Polish Navy (PN) soldiers and Maritime Unit of the Border Guard (MUBG) officers. Placing these two formations in one article stems from the fact that there are significant similarities in the tasks that they are responsible for [14,15]. There are similarities in their tasks and cooperation in the following areas:

1. Protection of the integrity of a maritime border and detection of symptoms that pose a threat to state security from the sea.
2. Protection of the Polish economic zone.
3. Protection of important transportation routes.
4. Participation in search-and-rescue operations at sea (SAR).
5. Participation in the ecological protection of Polish marine areas, the detection of pollution in the marine environment, and the detection of perpetrators.

In both formations, soldiers and officers have a statutory obligation to maintain their physical fitness at a level that ensures that they are able to perform their official duties. In both formations, soldiers and officers must participate in physical fitness tests once a year [16]. In order for cooperation between soldiers and officers to be at the same high level, they should be in similar physical condition. This is influenced by the level of physical activity undertaken and eating habits.

Considering the above, the purposes of this study were as follows:

1. Assessment of the physical activity level of PN soldiers and MUBG officers;
2. Comparative analysis of physical activity levels and metabolic rates between PN soldiers and MUBG officers;
3. Assessment of the nutrition of soldiers serving in units of the Polish Navy and MUBG officers in terms of the frequency of consumption of individual food groups.

The established hypotheses were as follows:

1. There are no differences in physical activity levels between PN soldiers and MUBG officers. The rationale for this hypothesis was the fact there is a large number of common tasks and that the two operate in a similar environment.
2. The model of nutrition adopted for both PN soldiers and MUBG officers is appropriate in terms of quality and quantity.

## 2. Materials and Methods

This study was conducted on 131 PN soldiers aged 33.1 ± 6.3 years old and 132 MUBG officers aged 38.6 ± 5.5 years old. Among the PN soldiers, 38% had higher education and the rest had secondary technical education, while 22% of MUBG officers had higher education and the rest had secondary education. All respondents were residents of cities with over 100,000 inhabitants.

A total of 98 questionnaires from PN soldiers and 94 from MUBG officers qualified for this study. The remaining questionnaires did not meet the reliability criteria. These questionnaires were returned only partially completed, or were not completed, or unreliable data were entered (e.g., 20 h of intensive work during 1 day of professional work).

### 2.1. Assessment of Physical Activity Level

In order to carry out this research study, the long version of the IPAQ was used. The introduction to the questionnaire included a definition of intensive and moderate physical activity in a way that was easy to understand for each participant of this study. Intensive physical activity is defined as vigorous physical effort which results in rapid, increased breathing and thus a higher heart rate. On the other hand, moderate physical activity is defined as an average effort, which results in slightly increased breathing and slightly higher heart rate [17].

The first part of the questionnaire was concerned with work-related physical activity. Three types of physical activity were distinguished: intense, moderate, and walking (marching). The second part is concerned with physical activity related to movement (walking, cycling). In the third section of this study, respondents were asked how much time they spend on physical activity related to work around the house, general household duties, and taking care of their families. The fourth part of the questionnaire was concerned with recreation, sports, and leisure-time physical activity. The fifth part included questions about time spent sitting down, divided into weekends and weekdays. Based on the number of days, time spent on a given physical activity, and the Metabolic Equivalent of Task (MET) ratio determined for a given physical activity, the total energy expenditure for each type of physical activity was calculated (1 MET = resting oxygen consumption of 3.5 mL/min/kg). Two people were trained to conduct the survey. The surveys were conducted in a training room. Before starting to fill out the IPAQ, the instructions for filling out the IPAQ were discussed. Trained people were present in the training room during the filling out of the questionnaires. If necessary, they answered individual questions in such a way as not to disturb other survey participants.

### 2.2. Assessment of Eating Habits

Eating habits were assessed based on a Food Frequency Questionnaire (FFQ) for 61 groups of food products. The respondents were asked to determine the frequency of consumption of each product during the last 12 months in accordance with the adopted scale: 1—never or almost never; 2—once a quarter or less often; 3—once a month or less often; 4—a few times a month; 5—once a week; 6—several times a week; 7—every day; 8—several times a day. The reported frequencies were expressed as daily frequencies (time/day) as follows: never or almost never—0.003 (1/365); once a quarter or less often—0.01 (4/365); once a month or less often—0.03 (1/30); a few times a month—0.08 (2.5/30); once a week—0.14 (1/7); several times a week—0.57 (4/7); every day—1; several times a day—2. The converted frequencies were also summed within the main food groups, which were identified as follows:

1. Fruits, vegetables, seeds of legumes and potatoes;
2. Cereal products;
3. Dairy products and eggs;
4. Meat products and fish;
5. Fats, nuts, and grains;
6. Sweets and snacks;
7. Soft drinks;
8. Alcoholic beverages.

This study was carried out in 2016–2020 as part of the National Health Program research project. Permission of the Bioethics Committee of the Military Institute of Hygiene and Epidemiology in Warsaw (Poland) was obtained for conducting the research (No 01/2016). All procedures were performed in accordance with the ethical standards as laid down in the 1964 Declaration of Helsinki and its later amendments or comparable ethical standards. All participants provided informed consent.

### 2.3. Statistical Analyses

The normality of the distribution of each variable was assessed with the use of the Shapiro–Wilk test. Data on PA were statistically analyzed with the use of the Statistica 13.3 software package (Statsoft, Krakow, Poland). Values were presented as means mediums and standard deviation (SD). Student’s *t*-test was implemented in order to determine the statistical significance of differences between the studied variables. Statistical analysis of dietary data was performed in the PS IMAGO PRO 9.0 program (IBM SPSS Statistics 29). The nonparametric Mann–Whitney U test was used to assess differences between the studied groups (PN soldiers and MUBG officers). The α-level of significance was set a priori at 0.05.

## 3. Results

### 3.1. Assessment of Physical Activity Level

Both of the studied groups showed similar levels of physical activity (no statistically significant differences). Over 80% of the soldiers and officers studied indicated high levels of PA. However, the MUBG officers indicated a higher percentage of people with high levels of PA and a lower percentage with low levels (Table 1).

MUBG officers showed a higher total MET value. Statistically significant differences were also found in the mean MET·min/week between the analyzed groups of study participants in the following categories (parts of the questionnaire): efforts related to professional work, efforts related to movement, and efforts related to housework (Table 2). The analysis of the total MET·min/week of intensive and moderate efforts and walking revealed that the differences between PN soldiers and MUBG officers occurred in intensive efforts (3726 vs. 4479, respectively). In the variables of moderate efforts and walking, no differences at a statistically significant level were found (3706 vs. 3887 and 4155 vs. 4301, respectively).

In terms of professional work, a greater total physical effort (at a statistically significant level) was indicated by Navy soldiers (Table 3). In terms of housework, the same was true for MUBG officers (Table 4).

However, statistically significant differences were found in the amount of time spent sitting down between the group of PN soldiers and the group of MUBG officers. On weekdays, MUBG officers spent more time sitting down (255 ± 159 min. vs. 298 ± 217 min.), while on weekends, PN soldiers spent more time sitting down (277 ± 183 min. vs. 214 ± 136 min).

### 3.2. Assessment of Eating Habits

Of the 61 food product groups analyzed, significant differences were found in the frequency of consumption of 17 products (Table 5). These differences concerned almost all groups (except group 3, i.e., dairy products and eggs). It was shown that MUBG officers consumed all types of fruits (0.78 vs. 0.59; *p* = 0.012) and stone fruits (0.31 vs. 0.18; *p* < 0.001), other tropical fruits (other than kiwi and citrus fruits) (0.18 vs. 0.10; *p* = 0.015), dried fruits (0.18 vs. 0.10; *p* = 0.012), and tomatoes (0.64 vs. 0.52; *p* = 0.022) significantly more often. Total vegetable consumption did not differ between the subjects. In the cereal product group, a significant difference was observed only for wholemeal products or products with grains, so-called dark bread—MUBG officers consumed these products significantly more often than PN soldiers (0.61 vs. 0.46; *p* = 0.048). Sausages (0.51 vs. 0.38; *p* = 0.0017) and red meat (0.27 vs. 0.19; *p* = 0.009) were consumed significantly more often by PN soldiers than by MUBG officers, similarly to all types of margarine (0.26 vs. 0.15; *p* = 0.002) and mayonnaise and dressings (0.18 vs. 0.14; *p* = 0.042). Moreover, PN soldiers used sugar to sweeten beverages (0.44 vs. 0.32; *p* = 0.009) and honey to sweeten food and beverages (0.26 vs. 0.19; *p* = 0.005) significantly more often. Significant differences were also observed in the consumption of products such as ice cream and pudding, salty snacks, energy drinks, sweetened sodas such as Fanta, Coca-Cola, Mirinda, and Sprite, and vodka and spirits. Attention should also be paid to the relatively low declared frequency of fruit and vegetable consumption in both study groups. Fruit was eaten at least once a day by only 28% (including 25% once a day and 3% several times a day) of PN soldiers and by 40% (including 30% once a day and 10% several times a day) of MUBG officers. However, vegetables were eaten at least once a day by only 43% (including 41% once a day and 2% several times a day) of PN soldiers and by 40% (including 32% once a day and 8% several times a day) of MUBG officers.

No correlations were found between physical activity variables and the nutritional assessment.

## 4. Discussion

The hypothesis adopted by the authors that there are no differences in PA levels between PN soldiers and MUBG officers was confirmed. Most likely, these results are justified by the similarity of some of the tasks that PN soldiers and MUBG officers are responsible for. The most important goal of performing these tasks is the defense of Poland’s maritime border. According to the authors, these results are not satisfactory, since, on average, one in five soldiers/officers do not achieve a high PA level. A high level is expected from soldiers and officers of uniformed services owing to the specific characteristics of the tasks they perform. The low (unsatisfactory) results of PA are especially concerning (8.2% PN soldiers; 4.3% MUBG officers). Clarifying this issue and taking preventive measures should be the priority for researchers and military decision-makers (commanders).

The analysis of MET results in the two examined groups provided interesting information. A higher MET·min/week was indicated by MUBG officers. This result was the effect of higher MET values found in two parts of the IPAQ, that is (1) movement efforts; (2) housework efforts. The biggest difference between PN soldiers and MUBG officers was in movement efforts (for the groups: 652 vs. 2842 MET·min/week, respectively). An in-depth analysis showed that this result was significantly influenced by data related to walking (for the groups: 418 vs. 1682 MET·min/week, respectively). Such a difference mainly results from the fact that MUBG officers perform their tasks on land and are required to travel on foot (patrolling an area), whereas PN soldiers perform patrolling with the use of vessels, on water.

The fourth part of the IPAQ provided interesting findings regarding physical recreation in leisure time. Based on the data from the IPAQ, it can be established that the involvement of PN soldiers and MUBG officers in leisure time activities is at a similar level and has a high MET·min/week. This shows that caring about physical fitness in their off-duty time is important for them. It also proves that people working in uniformed services are generally aware of the necessity to take care of their health and maintain a high level of PA, which has a positive effect on their level of physical fitness and the quality of their performance at work.

The authors conducted a comparative analysis between the results of this study and the results obtained by other authors. In general terms, the results indicate that there are no significant differences between the PA levels of PN soldiers and MUBG officers and soldiers or officers from other formations. For instance, a high level of PA was indicated by 81.6% of Air Force soldiers, 80.9% of flight crew soldiers, and 85.4% of airborne battalion soldiers [10,11]. In contrast, a low level was indicated by 8% of Air Force soldiers, 7.2% of flight crew soldiers, and 2.4% of airborne battalion soldiers.

As mentioned in the Introduction, it was challenging to find results of studies on the PA levels of soldiers in other armies with the use of the IPAQ. Finnish researchers surveyed recruits [12]. There is no point in comparing the PA of recruits with that of professional soldiers. However, it is worth mentioning that Jurvelin et al. found that about 25% of recruits showed an inadequate level of PA [12]. Furthermore, reviewing article databases with the focus on the Global Physical Activity Questionnaire (GPAQ) in uniformed services also failed to provide research findings on soldiers or Border Guard officers [18].

The study we presented constitutes another article on the level of PA of Polish soldiers and Border Guard officers. The results of this study complement existing data published after the completion of the National Health Program’s research project, as well as a scarce number of previous studies. These articles illustrated the general problem that, on average, almost one in fifteen soldiers/officers had a low level of physical activity according to the majority of the studies’ results. This leads to the realization that the measures undertaken by the military are not enough, despite the fact that they are broad-ranging and include promotional materials, lectures on healthy lifestyles, and guidance in the form of the EZAF educational platform for military service and face-to-face meetings [19]. It seems that the problem lies in the awareness of the surveyed soldiers/officers. Explaining this issue depends on researchers. It is difficult to address the question of whether this problem applies only to the Polish military service, since, as mentioned, there are no published results of studies by foreign researchers. Thus, it seems that the studies of Polish authors on the level of physical activity of soldiers/officers of uniformed services are of great value.

The proper nutrition of PN soldiers and MUBG officers should have a positive impact on their health but also consolidate healthy eating habits. The presented data on nutrition assessment show that there are significant differences in the structure of food consumption between PN soldiers and MUBG officers. These differences often concern products such as sausages and red meat, as well as margarine, mayonnaise, and sugar for sweetening drinks, which were consumed significantly more often by soldiers of the Polish Navy than by officers of the Border Guard. They are widely recognized as contributing to the development of diet-related lifestyle diseases. Similar results of research on the nutrition of Polish soldiers serving in various types of troops were obtained by Hyżyk et al., who showed an incorrect structure of nutrition of soldiers serving in selected military units of the Polish Armed Forces, which may consequently stimulate the emergence and development of obesity, atherosclerosis, and cardiovascular diseases and other lifestyle diseases [20]. Also, the results of research by Purvis et al. indicate the important role of dietary choices in maintaining high fitness of soldiers [21].

So far, tests of the physical activity and eating habits of PN soldiers have been carried out on a random basis, while such tests have not been carried out at all in the Maritime Unit Border Guard. This is the first comparative study providing the basis for determining the level of PA of PN soldiers and MUBG officers. The results of this study allow us to determine their PA, taking into account service specificity, and then develop an appropriate nutrition model that will cover their demand for energy and all nutrients and, at the same time, will have a preventive effect on diet-related metabolic diseases. We also propose that the training plans include mandatory theoretical classes on ways to improve physical fitness (various types of physical training) and the impact of individual types of training on the human body.

## 5. Conclusions

Overall, PN soldiers and MUBG officers presented high and moderate levels of physical activity. However, a few percent of soldiers and officers indicated low levels of PA, which is a disturbing phenomenon. Taking into account the demonstrated frequent consumption of high-energy and high-fat products, such as sausages and red meat, as well as margarine, mayonnaise and sugar, with the low frequency of fruit and vegetable consumption in both studied groups, it is advisable to conduct training among PN soldiers and MUBG officers in the field of health education regarding nutritional prevention of diet-related noncommunicable diseases and physically active lifestyle. We also believe that further research is needed to solve this problem and to undertake further educational activities of food service personnel of military units and Border Guard posts in the field of planning and implementation of rational nutrition and nutritional prevention of food-related diseases.

## Figures and Tables

**Table 1:** Level of physical activity of the surveyed PN soldiers and MUBG officers (percentage; number).

Subjects	Level of Physical Activity		
	High	Moderate	Low
PN soldiers	80.6% (79)	11.2% (11)	8.2% (8)
MUBG officers	88.3% (83)	7.4% (7)	4.3% (4)

**Table 2:** Mean MET·min/week associated with particular forms of physical effort performed by PN soldiers and MUBG officers (±SD).

Subjects	Efforts Related to Work	Efforts Related to Movement	Efforts Related to Housework	Efforts Related to Recreation	Total MET
PN soldiers	5607 ± 5412	652 ± 1804	2485 ± 2687	2843 ± 2687	11,588 ± 8451
MUBG officers	3912 ± 4818 *	2842 ± 3512 *	2933 ± 3415 *	2960 ± 3798	12,647 ± 8111 *

* differences between PN soldiers and MUBG officers at the significance level of *p* < 0.05.

**Table 3:** Metabolic rate (in MET·min/week) related to the performance of professional work by Navy soldiers and MUBG officers (±SD).

Subjects	MET Intensive	MET Moderate	MET Walking	Total MET
PN soldiers	1637 ± 2606	1556 ± 2272	2414 ± 2461	5607 ± 5564
MUBG officers	1652 ± 2767	895 ± 1726 *	1366 ± 1970 *	3912 ± 4818 *

* differences between PN soldiers and MUBG officers at the significance level of *p* < 0.05.

**Table 4:** Mean MET·min/week related to physical exertion performed during free time by Navy soldiers and MUBG officers.

Subjects	MET Intensive	MET Moderate	MET Walking	Total MET
PN soldiers	1324 ± 1520	1202 ± 1649	317 ± 614	2843 ± 2687
MUBG officers	1254 ± 2431	1338 ± 2034	368 ± 648	2960 ± 3798

**Table 5:** Frequency of product consumption by PN soldiers and MUBG officers.

Food Groups	PN Soldiers			MUBG Officers			*p*
	X	SD	ME	X	SD	ME	
**Fruits, vegetables, seeds of legumes, and potatoes**							
Fruits together—all types	**0.59**	**0.40**	**0.57**	**0.78**	**0.50**	**0.57**	**0.012**
Stone fruits	**0.18**	**0.23**	**0.08**	**0.31**	**0.33**	**0.14**	**<0.001**
Kiwi fruit and citrus	0.25	0.24	0.14	0.30	0.37	0.14	0.838
Other tropical fruits	**0.10**	**0.17**	**0.08**	**0.18**	**0.29**	**0.08**	**0.015**
Bananas	0.31	0.27	0.14	0.43	0.42	0.57	0.094
Apples and pears	0.40	0.36	0.14	0.48	0.35	0.57	0.093
Avocado	0.05	0.12	0.01	0.09	0.16	0.03	0.504
Olives	0.08	0.15	0.03	0.09	0.16	0.03	0.353
Dried fruits	**0.10**	**0.22**	**0.03**	**0.18**	**0.28**	**0.08**	**0.012**
Sweet fruit preserves and candied fruits	0.11	0.19	0.03	0.09	0.15	0.03	0.623
Vegetables—all types	0.72	0.37	0.57	0.75	0.47	0.57	0.882
Crucifers	0.31	0.25	0.14	0.33	0.35	0.14	0.665
Yellow–orange vegetables	0.43	0.34	0.57	0.41	0.35	0.57	0.504
Leafy green vegetables	0.29	0.33	0.14	0.34	0.35	0.14	0.205
Tomatoes	**0.52**	**0.33**	**0.57**	**0.64**	**0.38**	**0.57**	**0.022**
Vegetables: fresh cucumbers, squash, zucchini, pumpkin, eggplant	0.39	0.29	0.57	0.47	0.35	0.57	0.143
Root vegetables and others	0.41	0.31	0.57	0.38	0.32	0.57	0.382
Fresh and canned seeds of legumes	0.13	0.18	0.08	0.13	0.18	0.08	0.854
Dry seeds of legumes	0.10	0.14	0.08	0.07	0.13	0.03	0.238
Potatoes in various forms	0.47	0.32	0.57	0.43	0.33	0.57	0.285
Cereal products							
Wholemeal products or products with grains, so-called dark bread	**0.46**	**0.43**	**0.57**	**0.61**	**0.49**	**0.57**	**0.048**
Refined bread, so-called white bread	0.56	0.44	0.57	0.56	0.44	0.57	0.903
Unrefined groats, coarse	0.22	0.21	0.14	0.25	0.24	0.14	0.882
Refined cereal grain	0.18	0.20	0.08	0.24	0.24	0.14	0.164
Ready-to-eat breakfast cereal products	0.17	0.22	0.08	0.22	0.29	0.08	0.761
Dairy products and eggs							
Milk and milk drinks	0.55	0.45	0.57	0.54	0.47	0.57	0.617
Sweetened milk drinks	0.25	0.28	0.14	0.23	0.29	0.08	0.137
Cottage cheese	0.37	0.37	0.14	0.37	0.30	0.57	0.707
Flavored cottage cheese	0.14	0.20	0.08	0.15	0.23	0.03	0.087
Cheese	0.46	0.36	0.57	0.46	0.35	0.57	1.000
Eggs and egg dishes	0.37	0.27	0.57	0.40	0.27	0.57	0.383
Meat products and fish							
Sausages, different types	**0.51**	**0.37**	**0.57**	**0.38**	**0.31**	**0.57**	**0.017**
High-quality cold cuts	0.51	0.35	0.57	0.45	0.31	0.57	0.285
Sausage products and offal	0.17	0.23	0.08	0.11	0.16	0.08	0.132
Red meat	**0.27**	**0.26**	**0.14**	**0.19**	**0.27**	**0.08**	**0.009**
Poultry and rabbit	0.36	0.27	0.57	0.35	0.26	0.57	0.701
Wild game meat	0.03	0.07	0.01	0.04	0.10	0.01	0.413
Lean fish	0.14	0.18	0.08	0.11	0.13	0.08	0.178
Oily fish	0.11	0.24	0.08	0.10	0.14	0.08	0.664
Fats, nuts and grains							
Oil, all kinds	0.43	0.34	0.57	0.40	0.30	0.57	0.585
Butter, all types	0.54	0.42	0.57	0.61	0.49	0.57	0.563
Margarine, all types	**0.26**	**0.37**	**0.08**	**0.15**	**0.27**	**0.01**	**0.002**
Cream, sweet or sour cream, in foods or beverages	0.19	0.25	0.08	0.16	0.21	0.08	0.343
Other animal fats	0.11	0.21	0.03	0.06	0.13	0.01	0.086
Mayonnaise and dressings, i.e., salad dressings—all types	**0.18**	**0.23**	**0.08**	**0.14**	**0.21**	**0.08**	**0.042**
Nuts	0.22	0.32	0.08	0.25	0.29	0.08	0.481
Grains	0.13	0.20	0.03	0.17	0.25	0.08	0.075
Sweets and snacks							
Sugar to sweeten beverages	**0.44**	**0.53**	**0.14**	**0.32**	**0.51**	**0.01**	**0.009**
Honey to sweeten food and beverages	**0.26**	**0.34**	**0.08**	**0.19**	**0.31**	**0.03**	**0.005**
Chocolate, chocolate candies, and candy bars	0.21	0.25	0.08	0.36	0.39	0.14	0.064
Non-chocolate candies	0.12	0.19	0.03	0.10	0.19	0.03	0.066
Biscuits and cakes	0.14	0.18	0.08	0.22	0.26	0.08	0.120
Ice cream and pudding	**0.05**	**0.07**	**0.03**	**0.09**	**0.14**	**0.03**	**0.034**
Salty snacks	**0.09**	**0.13**	**0.08**	**0.09**	**0.18**	**0.03**	**0.040**
Soft drinks							
Fruit juices and fruit nectars	0.34	0.32	0.14	0.30	0.38	0.08	0.115
Vegetable juices and vegetable–fruit juices	0.14	0.26	0.08	0.22	0.39	0.08	0.458
Energy drinks	**0.10**	**0.21**	**0.03**	**0.02**	**0.11**	**0.00**	**<0.001**
Sweetened sodas such as Fanta, Coca-Cola, Mirinda, Sprite	**0.22**	**0.28**	**0.08**	**0.09**	**0.18**	**0.01**	**<0.001**
Alcoholic beverages							
Beer	0.13	0.16	0.08	0.18	0.28	0.08	0.979
Wine and cocktails	0.06	0.11	0.03	0.07	0.13	0.03	0.588
Vodka and spirits	**0.05**	**0.09**	**0.03**	**0.04**	**0.04**	**0.03**	**0.026**

Note: statistically significant differences are marked in bold.

## Data Availability

The data presented in this study are available on request from the corresponding author. The data are not publicly available due to privacy and ethical restrictions.
